# Rosacea is associated with conjoined interactions between physical barrier of the skin and microorganisms: A pilot study

**DOI:** 10.1002/jcla.23363

**Published:** 2020-05-17

**Authors:** Chao Yuan, Yafeng Ma, Yinjuan Wang, Xiuli Wang, Chunyan Qian, Didier Hocquet, Shuli Zheng, Sophie Mac‐Mary, Philippe Humbert

**Affiliations:** ^1^ Skin & Cosmetic Research Department Shanghai Skin Disease Hospital Tongji University School of Medicine Shanghai China; ^2^ Department of Dermatology Affiliated Hospital of Xuzhou Medical College Xuzhou China; ^3^ Amyway (China) R & D Center Shanghai China; ^4^ Department of Phototherapy Shanghai Skin Disease Hospital Tongji University School of Medicine Shanghai China; ^5^ Yuhang Branch of the Second Affiliated Hospital of Zhejiang University Hangzhou China; ^6^ Department of Infection Control University Hospital of Besançon France; ^7^ Skinexigence Besançon France; ^8^ Research and Studies Center on the Integument (CERT) Department of Dermatology Clinical Investigation Center (CIC INSERM 1431) University Hospital of Besançon Besançon France; ^9^ INSERM UMR1098 FED4234 IBCT University of Franche‐Comté Besançon France; ^10^ FONDATION Cheikh Khalifa Mohammed VI Casablanca Casablanca Morocco

**Keywords:** conjoined interaction, dysbiosis, microbiome, papulopustular rosacea, skin barrier function

## Abstract

**Background:**

Rosacea is a common condition characterized by transient or persistent central facial erythema, and often papules and pustules. Currently, the role of bacterium in the development and progression of rosacea remains controversial. This study aimed to investigate the difference in the physiological conditions and microorganisms between the lesional and non‐lesional areas of papulopustular rosacea.

**Methods:**

Twenty‐five French patients with papulopustular rosacea were enrolled in this pilot study. Each patient was subjected to clinical assessment, and the skin barrier function was tested in lesional and non‐lesional areas. In addition, samples from the lesional and non‐lesional areas were collected for bacterial culturing.

**Results:**

Of all subjects included in the study, a lower skin conductivity was measured in lesional areas than in non‐lesional areas (43.5 ± 12.4 vs. 57.2 ± 11.6 U, *P* < .05), and a higher transepidermal water loss (TEWL) value was found in lesional areas than in non‐lesional areas (17.2 ± 5.9 vs. 14.2 ± 4.1 g/(m^2^ h), *P* < .05). We found a lower TEWL in lesions in rosacea patients with bacterial dysbiosis than in those with bacterial balance (*P* < .05). In addition, there were significant differences in the skin conductivity and TEWL between lesional and non‐lesional areas in patients with bacterial dysbiosis (*P* < .001), and no significant differences were seen in patients with bacterial balance (*P* < .05).

**Conclusion:**

The results of the present study demonstrate that the physiological features of rosacea are closely associated with the interactions between the host and the microorganisms.

## 1 INTRODUCTION

Rosacea is a chronic skin disease characterized by transient or persistent central facial erythema, inflammatory papules and pustules, and often telangiectasia.^1^ Its main features include burning or stinging sensations, facial dryness, and edema.^2^ This disorder affects people of all ages but is most common in middle‐aged and older adults, with women being more frequently affected.^3^ Although several hypotheses have been proposed, the exact pathogenesis of rosacea remains unclear until now.^4^, ^5^, ^6^ Inflammation plays a prominent role in the pathophysiology of rosacea^7^; however, there is increasing evidence showing that skin barrier defects and dysregulation of the innate immune system are involved in the pathogenesis of rosacea.^8^, ^9^, ^10^ Microorganisms, such as *Demodex folliculorum*, *Staphylococcus epidermidis,* and *Propionibacterium acnes*, may also contribute to the pathogenesis of rosacea by stimulating the innate immune system, such as antimicrobial peptides (AMPs), Toll‐like receptors (TLRs), and other innate immune cells.^11^, ^12^, ^13^ There are some important links between skin physiological conditions and skin microbiota in many skin diseases, including rosacea.^14^, ^15^ However, it has not been fully demonstrated that whether microorganisms trigger rosacea, or dysbiosis is a response to changes in the skin microenvironment resulting from rosacea.

Skin physiological conditions could affect the microbial flora living on the face by affecting the skin microenvironment.^16^ Increased transepidermal water loss (TEWL) is reported to correlate with an increase in the skin disease severity,^17^ and skin is susceptible to *Demodex* mite infection in patients with rosacea, notably papulopustular rosacea (PPR).^18^ In addition, there is a large amount of evidence to suggest the links between human pathology and skin microbiota.^14^, ^15^ It is therefore plausible to hypothesize that a disrupted skin barrier may promote skin bacterial colonization, and that the damaged skin barrier and bacterial colonization may both trigger and aggravate skin diseases. In this study, we aimed to examine the associations of the density of bacterial colonization with the severity and skin barrier function in patients with rosacea.

## 2 MATERIALS AND METHODS

### 2.1 Subjects

PPR patients without any other systemic diseases were recruited. All diagnoses were confirmed by at least two dermatologists according to the National Rosacea Society diagnostic criteria,^19^ and each patient had at least three lesions (papules or pustules) on the face. Subjects undergoing topical treatment within 4 weeks prior to the enrollment or subjects on an antibiotic treatment scheme were excluded from the study. Finally, 25 PPR patients were enrolled in this study, and included 2 men and 23 women, with ages of 28‐60 years. The mean duration of rosacea was 11.8 ± 9 years, and none of the patients had taken medications during the past 3 months.

### 2.2 Clinical assessments

We utilized a broad subjective scoring system, based on a 4‐point scale, with 0 = normal, 1 = mild, 2 = moderate, and 3 = severe.^20^ The primary features included frequent flushing, persistent erythema, papules/pustules and telangiectasia. Other symptoms included burning or stinging, plaque, dryness, edema, ocular manifestations, or phymatous changes. Final assessments were made based on rosacea subtypes and subjects' self‐assessments.

### 2.3 Measurement of physical conditions

Skin samples were taken from the lesional (papules or pustules) and non‐lesional areas neighboring the lesions of the PPR patients, and TEWL and skin conductivity (which indirectly indicates skin water content) were measured by using the Tewameter TM210^®^ (Courage & Khazaka Electronic GmbH) and the Corneometer 820^®^ (Courage & Khazaka Electronic GmbH), respectively. Rosacea patients were at rest for at least 20 minutes in an environment‐controlled room: relative humidity of 40%‐60% and ambient temperature of 20°C‐22°C, prior to the measurements.

### 2.4 Bacterial culturing

Bacterial samples were taken from the lesional (at least three lesions for each patient) and the non‐lesional areas with two different dry sterile cotton swabs (VWR International, Inc), and each site was wiped 20 times with the same duration and pressure. Specimens were then incubated in Columbia agar supplemented with 5% of sheep blood (Thomas Scientific) at 35°C containing 5% CO_2_ for 5 days. Each bacterial colony was identified by matrix‐assisted laser desorption/ionization time of flight mass spectrometry (MALDI‐TOF MS) with a log value of ≥2 according to the manufacturer's recommendations (Bruker Daltonik GmbH).

### 2.5 Comparison of skin microbiome between the leisonal and non‐lesional areas

In this study, three methods were used based on the data of bacterial culturing, with two groups defined in each method. Method 1 was based on the presence of identical dominant microorganism in lesional and non‐lesional areas, and rosacea patients were assigned into the bacterial balance group (Group A) and bacterial dysbiosis group (Group B). Methods 2 and 3 were based on the presence of *P acnes* or *S epidermidis* in lesional and non‐lesional areas. In Method 2, *P acnes* as the dominant microorganism was assigned into Group A, while non‐*P acnes* assigned into Group B. In Method 3, *S epidermidis* as the dominant microorganism was assigned into Group A, while non‐*S epidermidis* assigned into Group B (Table 1).

**TABLE 1:** Three methods used to compare the skin microbiome between the lesional and non‐lesional areas

Method	Criteria of classification	Bacterial balance group (Group A)	Bacterial dysbiosis group (Group B)
1	Whether dominant microorganisms are totally consistent between the lesional and non‐lesional areas	The skin microbiome is totally consistent between the lesional and non‐lesional areas, indicating relative stable skin microbiome	There is a significant difference in the skin microbiome between the lesional and non‐lesional areas, indicating unstable skin microbiome (dysbiosis)
2	Whether *P acnes* is a dominant microorganism (*P acnes* is widely accepted as a protective bacterial colony)	*P acnes* is a dominant microorganism	*P acnes* is not a dominant microorganism
3	Whether *S epidermidis* is a dominant microorganism (*S epidermidis* is widely accepted as a protective bacterial colony)	*S epidermidis* is a dominant microorganism	*S epidermidis* is not a dominant microorganism

### 2.6 Ethics statement

This study was approved by the Institutional Ethics Review Committee. Written informed consent was obtained from all subjects participating in the study, following a detailed description of the purpose of the study. All experiments were performed in accordance with the Declaration of Helsinki.

### 2.7 Statistical analysis

Diversity indices for lesions and controls were calculated using vegan in R package version 3.3.0, and the distances were compared using Bray‐Curtis distance measurements. Calculations of Bray‐Curtis dissimilarities were done between datasets and hierarchical clustering using the R package. Differences between groups were tested for statistical significance with the Wilcoxon‐Mann‐Whitney rank sum test. All statistical analyses were performed using the statistical software SPSS version 16.0 (SPSS, Inc), and a *P* < .05 was considered statistically significant.

## 3 RESULTS

### 3.1 Skin conductivity and TEWL

Of all subjects enrolled in this study, a lower skin conductivity was detected in the lesional areas than in non‐lesional areas (43.5 ± 12.4 vs. 57.2 ± 11.6 U, *P* < .05), and a higher TEWL was seen in the lesional areas than in non‐lesional areas (17.2 ± 5.9 vs. 14.2 ± 4.1 g/(m^2^ h), *P* < .05).

There were no significant differences detected in the clinical assessment between groups A and B using methods 1, 2, or 3 (*P* > .05). In Method 1, a higher TEWL was found in lesions in Group A than in Group B (*P* = .016), and there were significant differences in both the skin conductivity and TEWL between the lesional and non‐lesional areas in Group B (*P* < .001) (Table 2). In Method 2, there were significant differences in both skin conductivity (*P* < .001) and TEWL (*P* = .01) between the lesional and non‐lesional areas in Group B, and there was a significant difference detected in the TEWL between lesional and non‐lesional areas in Group A (Table 3). In Method 3, significant differences were seen in the skin conductivity and TEWL between the lesional and non‐lesional areas in both groups A and B (*P* < .05) (Table 4).

**TABLE 2:** Comparison of physical barrier of the skin in Method 1

Method 1	Clinical assessment	Water content		TEWL	
		Lesional areas	Non‐lesional areas	Lesional areas	Non‐lesional areas
*P* value (A vs. B)	0.697	0.125	0.278	0.016	0.16
*P* value (A)		0.052		0.375	
*P* value (B)		< 0.001		<0.001	

Note

A, the bacterial balance group; B, the bacterial dysbiosis group.

**TABLE 3:** Comparison of physical barrier of the skin in Method 2

Method 2	Clinical assessment	Water content		TEWL	
		Lesional area	Non‐lesional area	Lesional area	Non‐lesional area
*P* value (A vs. B)	0.826	0.393	0.203	0.451	0.303
*P* value (A)		0.09		0.04	
*P* value (B)		<0.001		0.01	

Note

Group A, *P acne* is the dominant microorganism; Group B, non‐*P acne* is the dominant microorganism.

**TABLE 4:** Comparison of physical barrier of the skin in Method 3

Method 3	Clinical assessment	Water content		TEWL	
		Lesional area	Non‐lesional area	Lesional area	Non‐lesional area
*P* value (A vs. B)	0.857	0.643	0.504	0.377	0.394
*P* value (A)		0.04		0.011	
*P* value (B)		0.001		0.034	

Note

Group A, *S epidermidis* is the dominant microorganism; Group B, non‐*S epidermidis* is the dominant microorganism.

### 3.2 Composition and diversity of microbial communities

**FIGURE 1:**
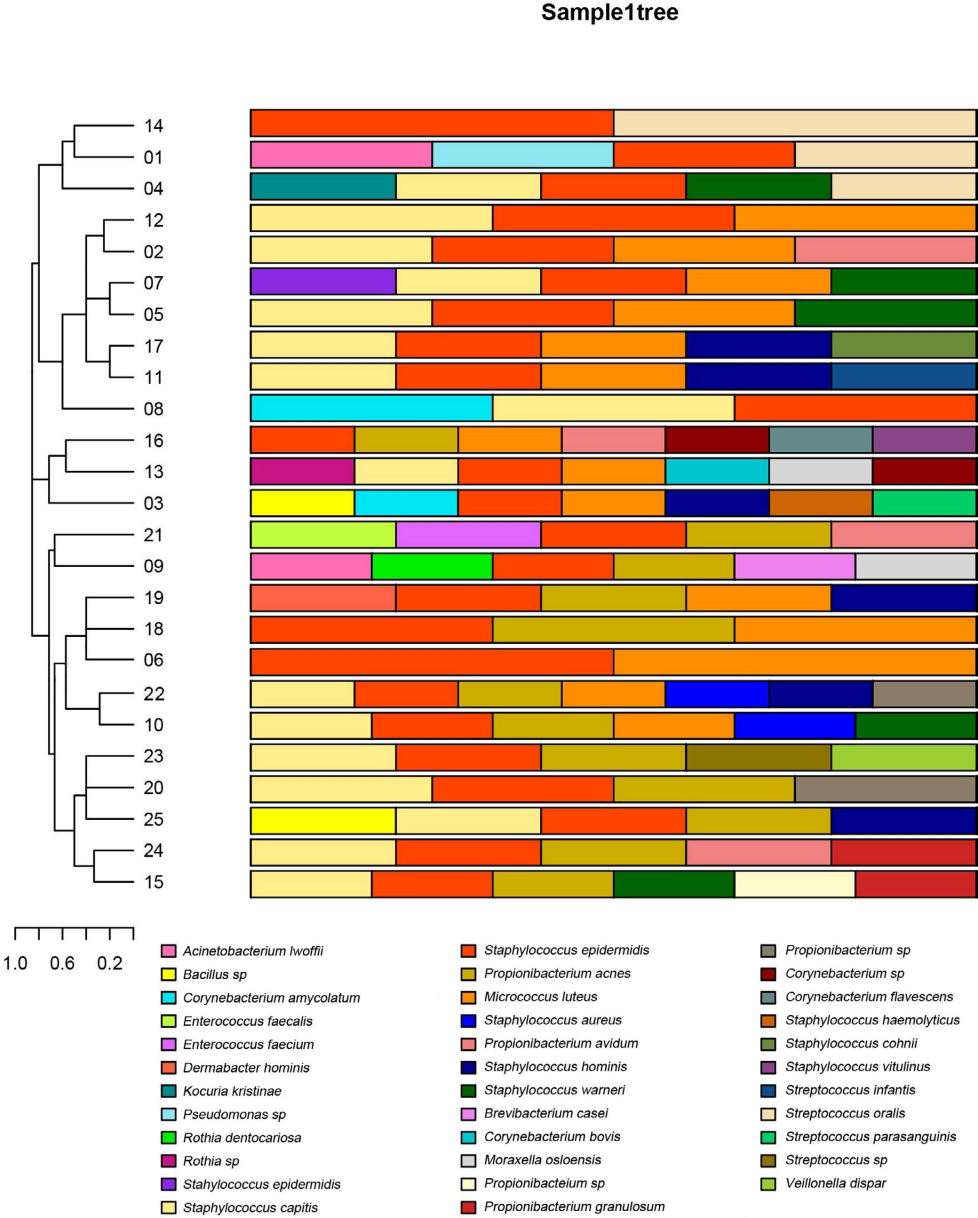
Relative abundance of the most predominant microorganisms in the lesions in 25 patients. Each color piece represents a species, and each color represents a sample of species abundance. Clustering is based on the similarity of species abundance, and various combinations between samples are made according to the strains of mesh, clustering, species and genera, to reflect the multiple samples of colony at the species level

The sequences of microorganisms were aligned with cmalign 1.0.2. On the left is the sample room based on the hierarchy of colony clustering analysis, and on the right is the histogram colony structure of sample. The similarities and differences of multiple samples in strains are presented using colors in Figure 1.

**FIGURE 2:**
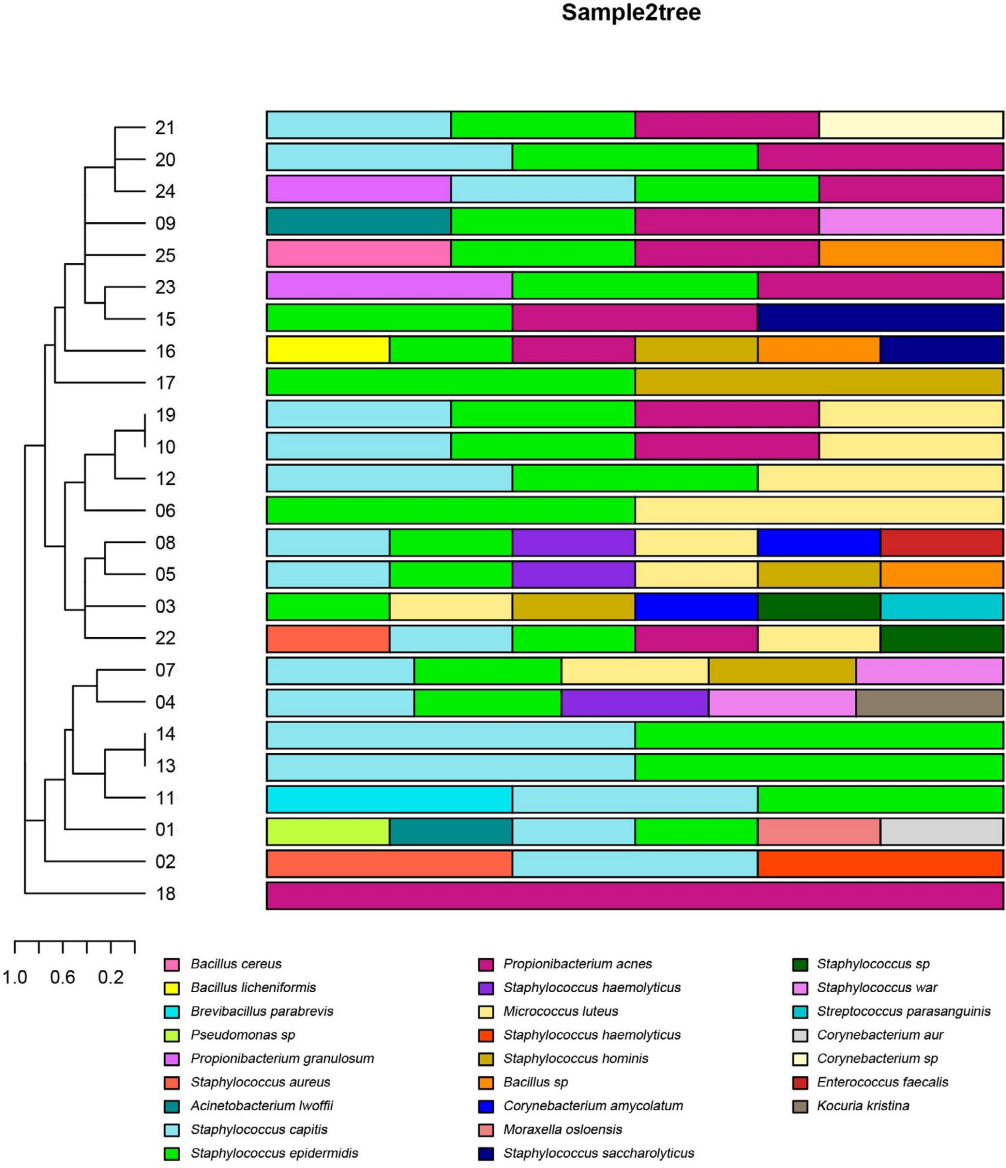
Relative abundance of the most predominant microorganisms in the control areas in 25 patients. Each color piece represents a species, and each color represents a sample of species abundance. Clustering is based on the similarity of species abundance, and different combinations between samples are made according to the strains of mesh, clustering, species and genera, to reflect the multiple samples of colony at the species level

There were 41 and 30 types of microorganisms identified in the lesional and non‐lesional areas, respectively, with 17 types of common microorganisms identified in both the lesional and non‐lesional areas (Figures 1 and 2). Comparison of the dominant microorganisms between the lesional and non‐lesional areas showed *S epidermidis*, *P acnes,* and *S capitis* as the three most prevalent microorganisms.

**FIGURE 3:**
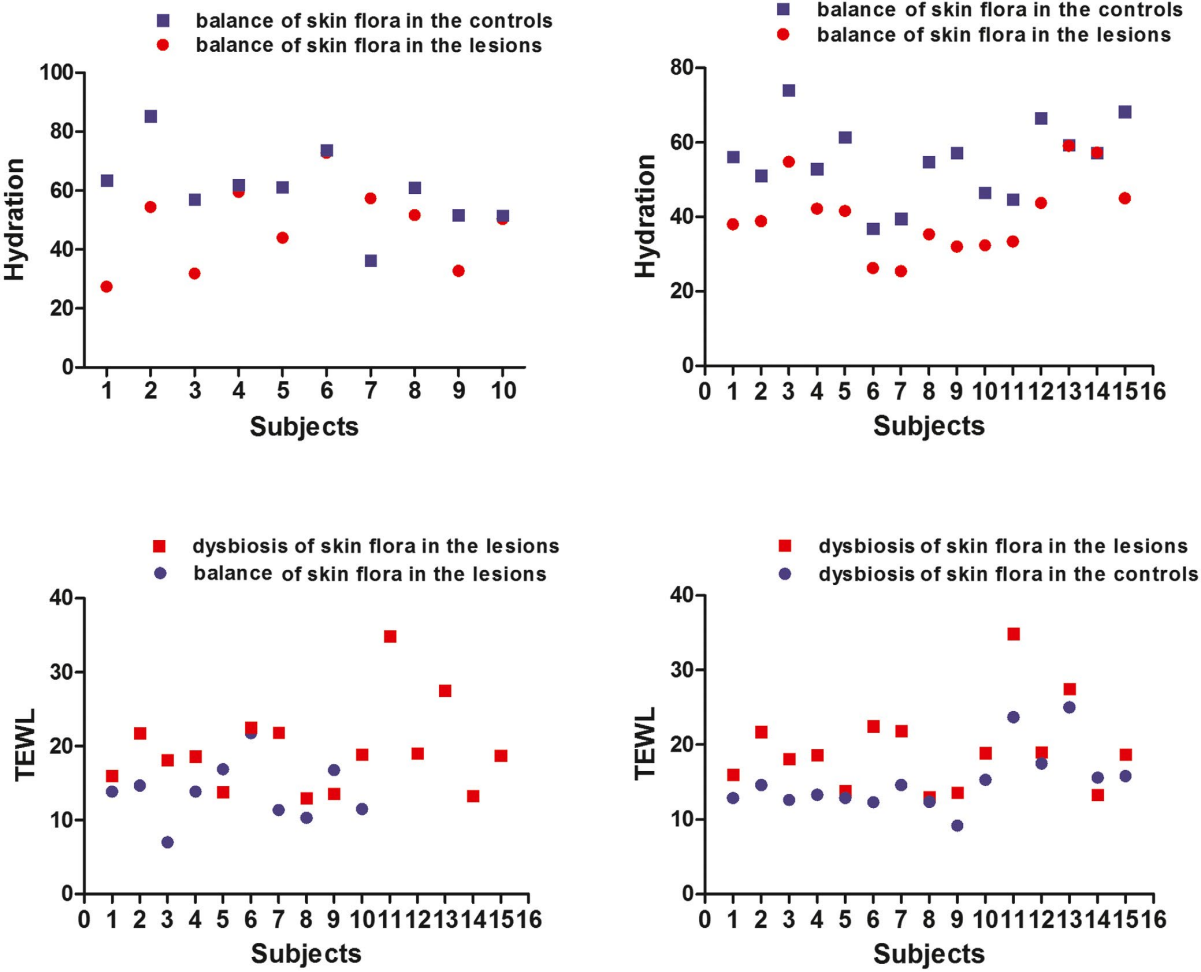
Dominant microorganism's difference (same species and different species) for physical barrier of the skin. A significant difference is found in skin physiological features (skin conductivity and TEWL) in the bacterial dysbiosis group between the lesional and non‐lesional areas (*P* < .001) compared with the bacterial balance group

Figure 3 shows the skin conductivity and TEWL in the lesional and non‐lesional areas in groups A and B, and there were significant differences in the skin physical parameters between the lesional and non‐lesional areas in Group A relative to Group B (*P* < .001).

## 4 DISCUSSION

Skin bacterial dysbiosis has been strongly linked to many skin diseases,^14^, ^21^, ^22^ and remarkable changes are observed in the bacterial diversity on patients' skin surface during the onset of acne, atopic dermatitis or hidradenitis suppurativa.^23^, ^24^, ^25^, ^26^, ^27^, ^28^ Therefore, a better understanding of the skin bacterial stability and diversity may provide new insights into diagnosis and treatment of skin diseases.

**FIGURE 4:**
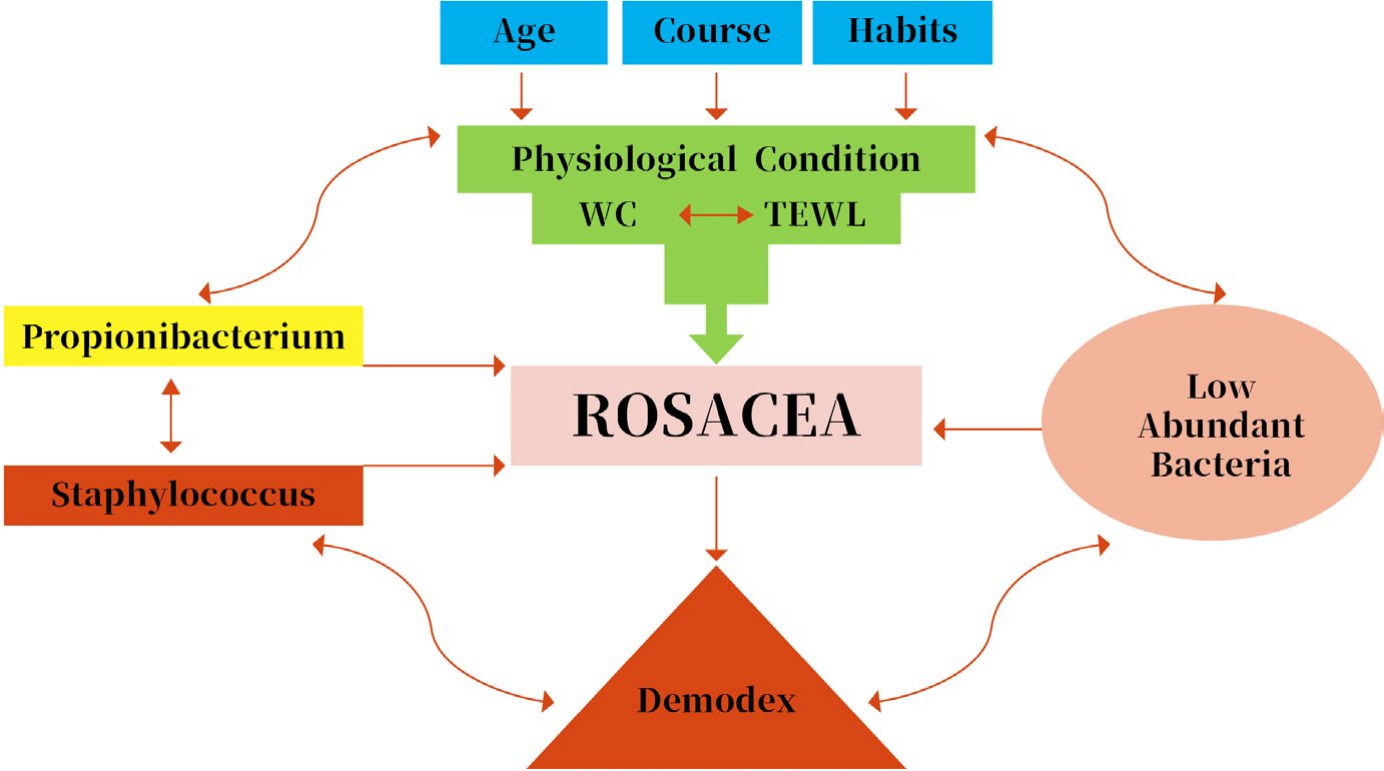
Overview of the relationships among rosacea, host demographics, physiological conditions, and microorganisms

The diagnostic criteria of rosacea include primary features, such as flushing erythema, permanent erythema, papules, pustules, telangiectasias, and other inflammatory lesions.^29^ Although the exact pathogenesis of rosacea is unknown, there are several factors implicated in the pathophysiology of rosacea.^5^, ^10^, ^11^ These influencing factors, which include age, medical course, sun, life habit, *Demodex,* and microorganisms, may affect skin physiological features such as water content and TEWL.^30^, ^31^, ^32^ The main microorganisms include *Propionobacterium*, *Staphylococcus,* and low‐abundant bacteria.^13^, ^31^ These stimuli induce episodes of flushing with progressive damage to the endothelium and angiogenesis, as well as inflammatory changes in the dermis with production of vasoactive substances, worsening of the vascular framework which will have repercussions in the epidermis.^8^ In addition, these influencing factors may interact with physiological features and microorganisms interact with each other (Figure 4). The role of microorganisms in the development of rosacea has been extensively investigated; however, the exact pathogenic role of microorganisms in rosacea has not been fully demonstrated until now and continues to be debated.^12^, ^13^

The primary function of human skin, one of the largest and most versatile organs in human body, is to protect the host from stimuli by external agents, including chemical, physical, and microbial factors.^14^ As the first barrier to environmental exposure, it is composed of dozens of distinctive and diverse microenvironments for colonization by a variety of microorganisms.^33^ Skin microbiota has been widely accepted to be of high importance for human health and well‐being.^16^ Multiple pathways and events that contribute to rosacea pathophysiology have recently been defined; however, the presence of a microorganism as a contributing agent remains controversial to date.^12^ The response of the microbes to inflammation and to the changes in microenvironments and macroenvironments is supposed to play a possible role in the pathophysiology of rosacea.^15^ The microbes inhabiting a given microenvironment are diversified, based on the suitability of these conditions for growth of each individual species.^15^ During normal skin homeostasis, the microbes inhabiting the microenvironment keep a balance; however, a disorder of the microenvironment may occur if factors affecting the growth or survival of microorganism change.^15^ In addition, changes in microbiota may be due to individual, environmental, or behavioral factors, such as age, gender, climate, hygiene, antibiotic consumption, humidity, temperature, pH, and lipid composition, which may cause dysbiosis.^34^ It is therefore of great importance to examine the correlation between microenvironments and rosacea, which may interact with each other.

Human skin provides a great living environment for the growth of microbes. *P acnes*, a major commensal of the human skin, colonizes the lipid‐rich sebaceous glands of the skin, and is presented as an opportunistic pathogen via bacterial seeding causing invasive infections.^35^ It has been shown that *P acnes* exhibits a strong proinflammatory activity and targets molecules involved in the innate cutaneous immunity, keratinocytes, and sebaceous glands of the pilosebaceous follicle.^35^ Our data indicated that *P acnes* correlated with the physiological features of rosacea, which was inconsistent with the previous study reporting no link between *P acnes* and rosacea.^36^ Further studies are required to examine the exact correlation between *P acnes* and rosacea.

*S epidermidis* is the most important member of the coagulase‐negative staphylococci and one of the most abundant colonizers of human skin.^33^ As a biofilm‐producing commensal found ubiquitously on human skin and mucous membranes, *S epidermidis* has been shown to be involved in rosacea.^33^ *S epidermidis* strains isolated from patients with rosacea have previously been shown to secrete more proteins and were more consistently beta‐hemolytic than those from control subjects.^33^ Its ability to cause disease is linked to its presence as a natural resident on human skin and its ability to attach and form biofilm on foreign bodies.^33^ In the study, *S epidermidis* was found to affect the skin barrier.

As a metabolically active structure that has adaptive features, stratum corneum may play a regulatory role in the process of inflammatory response.^35^ In the current study, we measured a lower water content and a higher TWEL in the lesions that in the control areas, which is consistent with previous studies demonstrating that the deficient stratum corneum has a low ability to attract and retain water.^35^ However, mild cleansers/moisturizers have been found to improve the stratum corneum barrier function and can help relieve symptoms.^35^

Prevention of rosacea is very important, which may be achieved by avoiding specific triggering factors, increasing skin water content, and decreasing TEWL.^36^ In the process of complex treatments, manifestations of impaired barrier function of the skin are observed, and the protection and restoration of the damaged stratum corneum are necessary.^25^ The treatment, with consideration of morphological and functional features of facial skin, may help improve the outcomes of therapy in patients with rosacea.^37^

Our study has several limitations. First, the study suffered from a small sample size. Second, no 16S rRNA gene‐based analysis was performed, and only two physiological features were investigated. Third, innate immunity, a main contributor to the pathogenesis of rosacea, was not noted. Fourth, previous studies have demonstrated the involvement of *Demodex* mites in the pathogenesis of rosacea,^18^, ^38^ and *Bacillus oleronius*, a Gram‐negative bacterium belonging to the genus *Bacillus*, is reported to colonize *Demodex* mites^39^, ^40^; however, we did not detect *Demodex* mites on the skin surface, but investigated the microorganisms isolated from the lesional and non‐lesional areas in patients with PPR. For the better understanding of the microbiology of rosacea, more studies are needed to help illustrate the mechanism of rosacea and contribute to providing more therapeutic approaches based on the controversial studies and opinions expressed in the literature.

## CONFLICT OF INTEREST

None declared.
